# Editorial: Unconventional animal models in infectious disease research, volume II

**DOI:** 10.3389/fcimb.2023.1225129

**Published:** 2023-06-14

**Authors:** Salma Younes, Nouran Zein, Shaden Abunasser, Layla Kamareddine, Natalia V. Kirienko, Gheyath K. Nasrallah

**Affiliations:** ^1^ Biomedical Research Center, Qatar University, Doha, Qatar; ^2^ Biomedical Sciences Department, College of Health Sciences, Qatar University, Doha, Qatar; ^3^ Department of BioSciences, Rice University, Houston, TX, United States

**Keywords:** animal models, infectious diseases, antimicrobial peptides, *Galleria mellollella*, host-pathogen interactions, Bordetella species, phagocytic predatory amoeba, *Dictyostelium discoideum*

Infectious diseases remain a serious health threat globally, an actuality that necessitates ongoing research in this field. Among different research modalities, the use of animal models to unravel host-pathogen interactions that contribute to infectious diseases pathogenesis is on the rise. By means of simple manipulation, these model organisms could be used to study host and microbial factors contributing to disease progression, host defensive strategies, and mechanisms of pathogenic dissemination. Recent advancements in the genetics discipline has further enhanced the utilization of both conventional and unconventional animal models in research. In this launched Research Topic, we provide valuable insight on the particular use of several less-common model organisms in infectious disease research.

Among several studies published in this Research Topic, Longhuan et al. explored how different *Bordetella* species interact with the phagocytic predatory amoeba *Dictyostelium discoideum* and revealed that the human pathogen *Bordetella bronchiseptica* has evolved to exploit this amoeba’s life cycle to propagate and spread. Interestingly, this trait was shared by a handful of other *Bordetella* species as well but not by all members of this genus, as the bird pathogen *B. avium* and the human-restricted species *B. pertussis* and *B. parapertussis* lack this ability and do not seem able to thrive in *D. discoideum.* Moreover, the genome-wide association study (GWAS) conducted for this paper identified 83 genes associated with the ability of *Bordetella* species to evade amoebic predation. Based on these findings, the authors speculate that the *Bordetella* species followed different evolutionary trajectories, with some increasingly adapting to single hosts while others remained more cosmopolitan in their spread. A better understanding of how *Bordetella* species resist amoeba predation may reveal promising targets for interventions to treat and prevent the diseases they cause.

Another area that has benefitted from the use of unconventional animal models is studying host-pathogen interactions, particularly to investigate diseases originating from pathogens that utilize insect vectors (such as mosquitoes) to spread and infect humans. Wide-spread adoption of intense insecticidal treatment in an apparently vain attempt to eradicate this vector has driven mosquitoes to develop resistance to this treatment approach. This has encouraged scientists to score for other vector control alternatives, one of which is targeting the immune system of insect vectors to minimize the ability of a pathogen to reside inside the insect host (Habtewold et al., 2008; Edgerton et al., 2020). Around the fundamentals of this notion, the study submitted by Morejon and Michel to our Research Topic aimed to investigate the role of antimicrobial peptides (AMPs) and Toll pathway components in the antimicrobial activity of *Anopheles gambiae* hemolymph by developing a novel assay for measuring AMP production via growth inhibition of bacteria on agar plates. AMPs are produced by innate immune genes, and their upregulation depends on signaling pathways like Toll and Imd. The authors of the paper leverage this useful, *ex vivo*, medium-throughput assay to show that bacterial challenge with *Micrococcus luteus* in *A. gambiae* drives *defensin 1*-mediated AMP expression. Interestingly, they determined that a sterile challenge (i.e., sterile water) primed the innate immune system for a stronger reaction, suggesting that the host is engaging in some sort of immune surveillance for breaches of its body cavity. A more detailed understanding of the regulation of the mosquito immune system’s antimicrobial activity could lead to the development of innovative strategies for minimizing host infection and disease transfer.

Another insect gaining popularity for infectious disease research is the lepidopteran *Galleria mellonella* also known as the greater wax or the honeycomb moth. This organism has several advantages, including its moderate conservation of innate immune pathways, comparative experimental simplicity, and low cost (Bismuth et al., 2019). In one of the studies submitted to our Research Topic, Felix et al. investigated the potentiality of α-mangostin, a natural xanthone molecule, to kill methicillin-resistant *Staphylococcus aureus* (MRSA) persisters and to disrupt established biofilm and prevent its future formation. The authors found that the minimum inhibitory concentration for α-mangostin in persister cells was 2 µg/ml and that it quickly permeabilized the cell membrane. Although α-mangostin was found to be non-toxic to liver-derived HepG2 and lung-derived A549 cells at 8 times this concentration, it also lysed 50% of human erythrocytes. *In vivo*, α-mangostin increased the survival rate of *Galleria mellonella* larvae infected with MRSA persisters by up to 75% after 120 hours. This study presented *Galleria mellonella* as a potential model organism for studying infectious diseases. However, it is also a cautionary tale, reminding us that studies in closer organisms are often needed to more thoroughly assess compound toxicity before considering systemic administration. Despite this, the authors foresee some aptitude for α-mangostin as a treatment option for MRSA-related topical infections.

Another published article in our launched Research Topic by Ménard et al., reviewed the broader use of *Galleria mellonella* as a model for bacterial infection, focusing on immunological and microbiological aspects. The authors emphasized virulence and new therapies for many important pathogenic bacteria and discussed directions for further improvement of this model. This paper, along with other recent reviews (Junqueira, 2012; Asai et al., 2023) illustrate *Galleria mellonella*’s significant potential in host-pathogen interaction studies.

Honeybees are also emerging as a potential model organism in infectious disease research. In one of the articles submitted to our Research Topic, Chang et al. investigated the effect of *Escherichia coli* LF82, an inflammatory bowel disease-associated bacterium, on the cognitive abilities of honeybees. Exposure to LF82 increased gut permeability, impaired learning and memory, and reduced tryptophan metabolism in the gut. The study also examined transcriptome changes in the honeybee brain and found that LF82 colonization altered the expression of 255 host genes. The study suggested that LF82 can induce enteritis-like manifestations and cognitive impairment through escape of gut metabolites, causing transcriptional changes in the brain. This study expands our understanding of the link between gut microbiota and neurological disorders, a field of increasing interest (Cryan et al., 2019; Zou et al., 2023).

In conclusion, the articles highlight significant progress in infectious disease research using unconventional animal models, leading to exciting discoveries and therapies. We anticipate increased utilization of these models, driving faster progress and deeper insights into infectious diseases. Exploring unconventional animal models will advance our knowledge and interventions.

## Author contributions

SY, NZ, and SA drafted the editorial. NK and LK revised the editorial critically for important intellectual content. GN: designed the work, revised the editorial critically for important intellectual content and provided final approval of the version to be published. All authors contributed to the article and approved the submitted version.
